# Um Novo Escore de Risco Baseado em Aprendizado de Máquina (Machine Learning) em Pacientes com Insuficiência Cardíaca Aguda: O Escore ML-HF

**DOI:** 10.36660/abc.20250136

**Published:** 2025-12-17

**Authors:** Matheus Bissa Duarte Ferreira, Jorge Tadashi Daikubara, Gustavo S. Pereira da Cunha, Rafael Moretti, Jessica Tamires Reichert, Lucas Müller Prado, Raphael Henrique Déa Cirino, Sidney C Smith, Fábio Papa Taniguchi, Andrei C. Sposito, Odilson M. Silvestre, Wilson Nadruz, Miguel Morita Fernandes-Silva

**Affiliations:** 1 Universidade Federal do Paraná Curitiba PR Brasil Universidade Federal do Paraná, Curitiba, PR – Brasil; 2 University of North Carolina At Chapel Hill Chapel Hill North Carolina EUA University of North Carolina At Chapel Hill, Chapel Hill, North Carolina – EUA; 3 Hospital do Coração São Paulo SP Brasil Hospital do Coração (HCor), São Paulo, SP – Brasil; 4 Universidade Estadual de Campinas Campinas SP Brasil Universidade Estadual de Campinas, Campinas, SP – Brasil; 5 Universidade Federal do Acre Rio Branco AC Brasil Universidade Federal do Acre, Rio Branco, AC – Brasil

**Keywords:** Insuficiência Cardíaca, Aprendizado de Máquina, Morte

## Abstract

**Fundamento:**

Os escores de avaliação prognóstica convencionais muitas vezes não têm desempenho suficiente para prever a mortalidade em pacientes com insuficiência cardíaca aguda (ICA).

**Objetivo:**

Desenvolver e validar um escore prognóstico baseado em aprendizado de máquina ( *Machine Learning - escore ML-HF)* para prever morte hospitalar em pacientes com ICA e comparar seu desempenho com os principais escores tradicionais.

**Métodos:**

Pacientes admitidos por ICA em hospitais brasileiros do “Programa de Boas Práticas em Cardiologia” de 2016 a 2022 foram incluídos. Dados clínicos, resultados laboratoriais e o questionário de Qualidade de Vida da Organização Mundial da Saúde ( *World Health Organization Quality of Life)* WHOQOL-Bref foram coletados na admissão hospitalar. O desfecho foi óbito hospitalar. O modelo foi treinado usando 70% das admissões (conjunto de treinamento) e validado com os 30% restantes (conjunto de teste). A área sob a curva ROC (AUC) do escore ML-HF foi comparada com a AUC dos escores tradicionais *Acute Decompensated Heart Failure National Registry* (ADHERE) e *Get With the Guidelines–Heart Failure* (GWTG-HF). O nível de significância foi de p < 0,05.

**Resultados:**

Foram incluídos mil cento e cinquenta e sete pacientes hospitalizados por ICA. As cinco variáveis mais importantes do escore ML-HF foram: Qualidade do Domínio de Saúde Física (WHOQOL-BREF), sódio sérico, ureia sérica, creatinina sérica e pressão arterial sistólica na admissão hospitalar. No conjunto de teste, o escore ML-HF apresentou calibração de modelo adequada (valor de p do teste de Hosmer-Lemeshow = 0,056) e discriminação (AUC = 0,722 [IC95%, 0,661-0,783]), que foi superior aos escores GWTG-HF (AUC = 0,616 [IC95%, 0,529-0,702; p = 0,014]) e ADHERE (AUC = 0,601 [IC95%, 0,511-0,691; p = 0,006]).

**Conclusão:**

Desenvolvemos e validamos um escore usando aprendizado de máquina para prever morte hospitalar em pacientes com ICA, que superou os escores tradicionais.

## Graphical abstract

**Figura Central:**
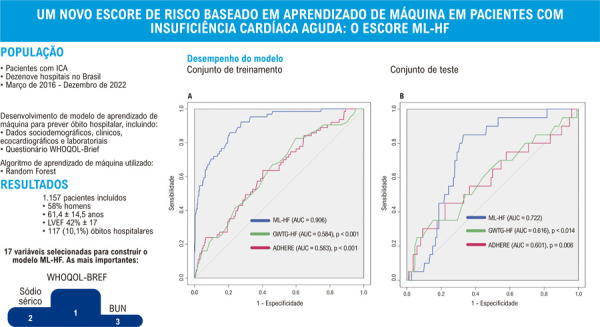
Um Novo Escore de Risco Baseado em Aprendizado de Máquina (Machine Learning) em Pacientes com Insuficiência Cardíaca Aguda: O Escore ML-HF

## Introdução

A insuficiência cardíaca (IC) afeta 25 milhões de pessoas em todo o mundo e apresenta altas taxas de mortalidade e hospitalização. No Brasil, ocorrem aproximadamente 250.000 internações hospitalares por IC por ano, resultando em uma taxa de mortalidade hospitalar de 10,9%.^1^

As ferramentas de classificação de risco ajudam a direcionar recursos e medidas intensivas para pacientes de alto risco. No entanto, estimar o prognóstico dos pacientes na admissão ou durante a hospitalização tem sido desafiador no cenário da IC. Estudos anteriores desenvolveram escores, como o *Acute Decompensated Heart Failure National Registry* (ADHERE) e o *Get With The Guidelines-Heart Failure* (GWTG-HF), que podem ser derivados de umas poucas variáveis laboratoriais e clínicas, para predizer a mortalidade hospitalar nesses pacientes.^2 , 3^ Infelizmente, esses escores têm precisão abaixo do ideal e têm sido subutilizados na prática clínica.^4 , 5^

Uma alternativa a essas abordagens convencionais são os modelos baseados em aprendizado de máquina ( *Machine Learning* - ML), que têm sido estudados nos últimos anos para o manejo de IC.^6 , 7^ A capacidade dos algoritmos de ML de analisar de forma mais profunda, multidimensional e não linear uma variedade de variáveis aumenta seu potencial para melhorar os modelos de estratificação de pacientes.^7^ Modelos baseados em ML foram construídos em pacientes com IC crônica e aguda usando registros médicos eletrônicos (RME), registros e ensaios clínicos.^8^ Esses modelos parecem ter precisão superior à dos escores tradicionais ADHERE e GWTG-HF.^9^ No entanto, a maioria desses estudos foi desenvolvida e validada em países de alta renda. Diferenças na população de pacientes, determinantes sociais e prestação de cuidados de saúde podem impedir a implementação desses modelos em países de baixa e média renda (PBMR). De fato, já foi demonstrado que a capacidade dos modelos de ML de prever desfechos de IC varia significativamente entre diferentes subgrupos étnicos e grupos socioeconômicos.^10^

Portanto, desenvolvemos e validamos um algoritmo baseado em ML para prever mortes hospitalares em pacientes com IC aguda, utilizando um amplo estudo de coorte em hospitais públicos no Brasil. Comparamos ainda o algoritmo final baseado em ML com os modelos ADHERE e GWTG.

## Métodos

### População

Trata-se de uma análise do programa “Boas Práticas em Cardiologia (BPC)”, adaptado da iniciativa “ *Get With The Guidelines* ” (GWTG) para melhorar a qualidade do atendimento cardiovascular e os desfechos dos pacientes com síndrome coronariana aguda, IC e fibrilação atrial em hospitais públicos brasileiros.^11^ Resumidamente, foram selecionados hospitais públicos terciários em todas as cinco macrorregiões brasileiras desde janeiro de 2016.^12^ Esses hospitais foram incluídos em um estudo quase experimental que implementou uma intervenção em nível institucional para melhorar a adesão às diretrizes e que avaliou seu impacto nas medidas de desempenho institucional. Dados sociodemográficos, clínicos, laboratoriais e ecocardiográficos foram coletados durante a admissão dos pacientes. Os pacientes também foram solicitados a preencher o questionário de Qualidade de Vida da Organização Mundial da Saúde (WHOQOL-Bref), que é um instrumento de 26 itens que consiste em quatro domínios: saúde física (7 itens), saúde psicológica (6 itens), relações sociais (3 itens) e saúde ambiental (8 itens). Os itens do WHOQOL-Bref consistem em escalas Likert de 5 pontos que, combinadas, fornecem cada domínio de saúde. O procedimento para calcular o WHOQOL-Bref foi descrito em detalhe em outro lugar.^13^Como parte do programa BPC, todos os participantes foram acompanhados com visitas na alta, um mês e seis meses. Este estudo foi aprovado pelo comitê de ética do Centro Coordenador (número 48561715.5.1001.0060) e de cada hospital participante. Todos os participantes assinaram um termo de consentimento livre e esclarecido para participação no estudo.

### Critérios de inclusão e exclusão

Para esta análise, foram incluídos pacientes com 18 anos ou mais internados com diagnóstico primário de IC aguda (códigos CID-10 I50; I50.0; I50.1 ou I50.9) entre 14/03/2016 e 07/12/2022 em qualquer hospital participante do programa BPC, que concordaram em participar do estudo e assinaram o termo de consentimento livre e esclarecido conforme ilustrado na Figura Central . Os participantes precisavam ter ficado hospitalizados por pelo menos 24 horas para serem elegíveis.

Excluímos pacientes transferidos para outro hospital ou com estado vital ausente na alta. Pacientes que estavam em lista de espera para transplante cardíaco ou em uso de dispositivo de assistência ventricular (DAV) também foram excluídos. Também excluímos pacientes com dados ausentes para as variáveis selecionadas do modelo (veja a seleção de variáveis abaixo).

Para construir e validar o modelo, dividimos o conjunto de dados em duas coortes: Coorte 1, pacientes internados de 16/01/2016 a 15/10/2021, e coorte 2, pacientes internados de 16/10/2021 a 07/12/2022. A coorte 1 foi utilizada nos processos de seleção de variáveis, construção do modelo e validação. A coorte 2 foi utilizada apenas para validação externa (veja Análise estatística para obter detalhes).

## Desfecho

O desfecho foi morte por qualquer causa durante a hospitalização índice.

### Seleção de variáveis

Utilizamos todas as variáveis na admissão no modelo preditivo, que incluiu dados sociodemográficos, clínicos, laboratoriais e ecocardiográficos, juntamente com os escores derivados das respostas do questionário WHOQOL-Bref. Primeiramente, excluímos variáveis com mais de 40% de dados ausentes.^14^ Veja a tabela Tabela Suplementar S1 para todas as variáveis e respectivas taxas de dados ausentes. Em seguida, aplicamos o método de ML “Boruta” no conjunto de treinamento (veja abaixo) para detectar automaticamente as variáveis mais relevantes que foram selecionadas para o algoritmo de treinamento. O método Boruta gera uma distribuição de probabilidade binomial do número de vezes que uma variável excede a importância de uma variável fictícia criada, que tem um valor aleatório (não preditivo).^15^ As variáveis foram selecionadas se tivessem um valor de p < 0,05 na cauda superior da distribuição de probabilidade. Durante esse processo de seleção, aplicamos um método de imputação K-vizinhos mais próximos, selecionando os K vizinhos mais próximos (K = 5) com dados completos com base em uma distância de Gower e imputando o valor médio da variável completa aos vizinhos com valores ausentes.^16^

**Figura 1:**
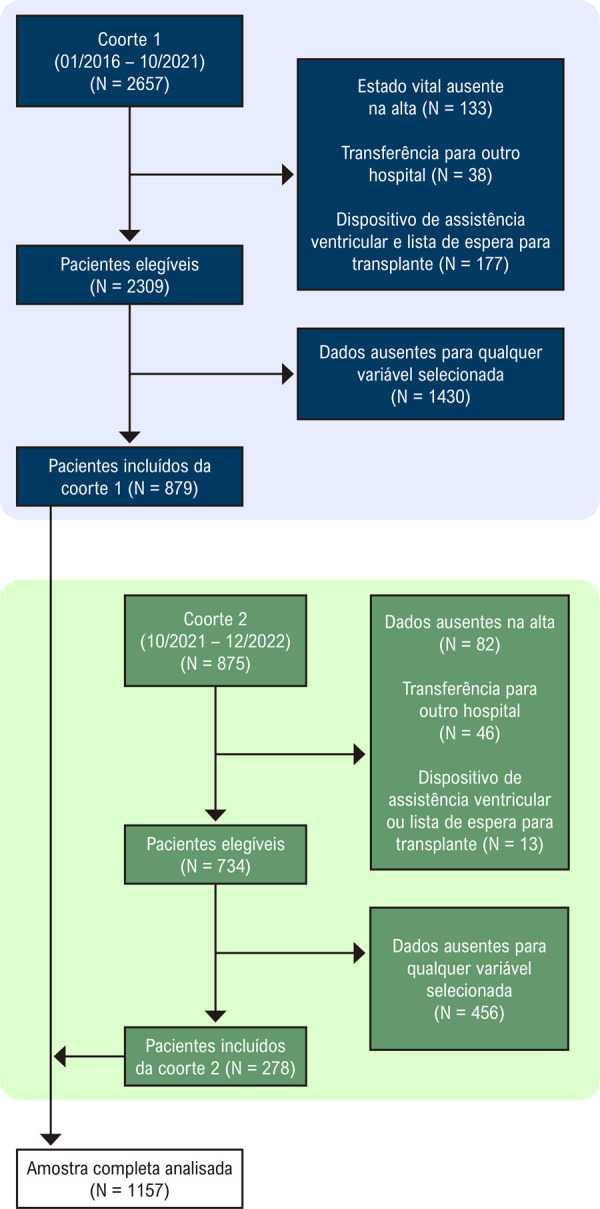
– Fluxograma de pacientes incluídos na análise.

Após a seleção das variáveis mais relevantes, elas foram utilizadas para construir o algoritmo de ML no banco de dados não imputado. A Figura S1 ilustra o processo de seleção de variáveis ( Figura Suplementar S1 ). Realizamos uma Análise de Casos Completos, incluindo apenas pacientes com dados completos para as variáveis selecionadas. Assim, pacientes com dados ausentes para qualquer uma das variáveis selecionadas foram excluídos ( Figura 1 ).

### Análise estatística

#### Estatísticas descritivas

As variáveis contínuas foram avaliadas quanto à normalidade por meio da inspeção visual dos histogramas, assimetria e curtose, e pela aplicação do teste de Kolmogorov-Smirnov. Os dados são apresentados como média ± desvio-padrão ou mediana (percentil 25 - percentil 75), conforme apropriado. As comparações entre os grupos foram realizadas utilizando o teste- t de Student não pareado para variáveis com distribuição normal e o teste U de Mann-Whitney para variáveis com distribuição anormal. As variáveis categóricas foram expressas como contagens e porcentagens e comparadas utilizando o teste exato de Fisher.

#### Desenvolvimento e validação de modelos

**Figura 2:**
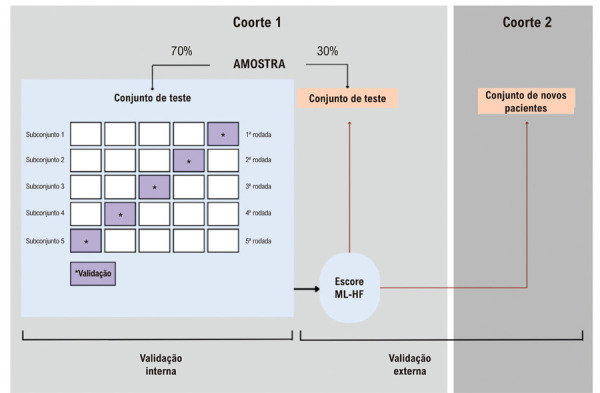
– Fluxograma ilustrando a validação interna e externa do modelo de escore ML-HF.

A coorte 1 foi dividida aleatoriamente em conjuntos de treinamento (70%) e teste (30%). Este procedimento foi realizado por meio de amostragem aleatória gerada por computador, sem reposição, garantindo igual probabilidade de seleção de cada paciente. O conjunto de treinamento foi usado para treinamento do modelo e validação interna. O conjunto de teste combinou 30% da coorte 1 com a coorte 2 completa e foi usado para validação externa. Uma análise de sensibilidade foi realizada usando apenas a coorte 2 como conjunto de teste. Este processo é ilustrado na Figura 2 .

#### Seleção de modelos

Utilizamos um método de validação cruzada de 5 etapas para selecionar o algoritmo de ML mais adequado. Este método consiste em subdividir o conjunto de treinamento em 5 grupos aleatórios. Quatro desses subconjuntos foram usados para treinar o modelo e o subconjunto restante para testá-lo. Trata-se de um processo iterativo que ocorre 5 vezes, alterando o subconjunto de teste, conforme ilustrado na Figura 2 . O algoritmo com a melhor AUC média foi escolhido como modelo final.

#### Desempenho de modelo

Para avaliar a discriminação do modelo, construímos as curvas ROC ( *Receiver Operating Characteristic* ) e comparamos a área sob a curva ROC (AUC) usando a morte hospitalar como desfecho. A calibração do modelo foi avaliada plotando a probabilidade prevista versus a observada de morte hospitalar em decis e calculando o teste de qualidade de ajuste de Hosmer-Lemeshow. Um valor de p < 0,05 indicou ajuste inadequado dos dados, enquanto valores de p mais altos indicaram calibração adequada.

#### Comparação com escores tradicionais

Os escores ADHERE e GWTG-HF foram calculados para cada paciente. Como o escore GWTG-HF não fornece diretamente as probabilidades de morte, derivamos uma regressão logística de seus resultados numéricos.^2 , 3^ Comparamos a AUC-ROC entre os escores usando o teste de DeLong nos conjuntos de treinamento e teste.^17^

Seguimos a diretriz de Relatório Transparente de um Modelo de Predição Multivariável para Prognóstico ou Diagnóstico Individual (TRIPOD) para conduzir nossa pesquisa e escrever este artigo.^18^

Todas as análises e treinamento do modelo foram conduzidos usando o software R. Consideramos estatisticamente significativos valores de p < 0,05.

## Resultados

De janeiro de 2016 a dezembro de 2022, um total de 3.532 pacientes com IC aguda foram incluídos no programa BPC. Após a exclusão daqueles com DAV, na lista de espera para transplante ou sem estado vital na alta, 3.043 foram considerados elegíveis para o estudo (2.309 na coorte 1 e 734 na coorte 2, como visto na Figura 1 ).

Das 113 variáveis disponíveis na admissão, nove foram excluídas devido à ausência de mais de 40% de dados (Tabela Suplementar S1). O algoritmo de Boruta foi aplicado a 70% dos pacientes (n = 1.616) selecionados aleatoriamente da coorte 1, resultando em 17 variáveis selecionadas para construir o modelo de ML.

Dos 3.043 pacientes elegíveis, 1.157 foram incluídos na análise final devido à disponibilidade completa de dados para todas as 17 variáveis. O conjunto de treinamento consistiu em 621 pacientes, o que representou 70% dos 879 indivíduos da coorte 1, enquanto o conjunto de teste consistiu em 536 pacientes, compreendendo os 258 pacientes restantes da coorte 1 e 278 pacientes mais da coorte 2.

A taxa de mortalidade hospitalar foi de 10,1%, com um total de 117 óbitos entre os 1.157 pacientes examinados. A idade média desses pacientes foi de 61 ± 15 anos e a fração de ejeção do ventrículo esquerdo (FEVE) foi de 42 ± 17% e 58% eram do sexo masculino ( Tabela 1 ). A Tabela 2 exibe as variáveis selecionadas no escore ML-HF e aquelas usadas nos escores ADHERE e GWTG para comparação.

**Tabela 1:** – Características basais dos pacientes incluídos

Parâmetros	Alta com vida (n=1040)	Morte hospitalar (n=117)	Valor-p
**Idade, anos**	61,3 ± 14,5	61,6 ± 14,4	0,829
**Sexo feminino, n(%)**	424 (40,8)	53 (45,3)	0,398
**Raça branca, n(%)**	674 (64,8)	78 (66,7)	0,766
**Educação, n(%)**			
Analfabeto	84 (8,1)	9 (7,7)	0,242
Ensino fundamental incompleto	399 (38,4)	47 (40,2)	
Ensino fundamental completo	148 (14,2)	26 (22,2)	
Ensino médio incompleto	72 (6,9)	8 (6,8)	
Ensino médio completo	225 (21,6)	20 (17,1)	
Ensino superior incompleto	30 (2,9)	2 (1,7)	
Ensino superior completo	82 (7,9)	5 (4,3)	
**Renda média, n(%)**			
≤ 1 salário-mínimo	721 (69,3)	97 (82,9)	0,009
**Fumante, n(%)**			
Não	520 (50)	56 (47,9)	0,889
Atual	438 (42,1)	52 (44,4)	
Passado	82 (7,9)	9 (7,7)	
**Alcoólatra, n(%)**			
Não	654 (62,9)	80 (68,4)	0,200
Atual	246 (23,7)	28 (23,9)	
Passado	140 (13,5)	9 (7,7)	
**Etiologia da IC, n(%)**			
Isquêmica	200 (19,2)	22 (18,8)	< 0,001
Doença de Chagas	95 (9,1)	13 (11,1)	
Doença valvar	123 (11,8)	30 (25,6)	
Outro	622 (59,8)	52 (44,4)	
Prótese valvar, n(%)	338 (32,5)	52 (44,4)	0,013
Insuficiência renal crônica, n(%)	152 (14,6)	27 (23,1)	0,024
Hipotireoidismo, n(%)	77 (7,4)	19 (16,2)	0,002
FEVE, %	41,7 ± 17,1	41,90 ± 18,5	0,911
Diâmetro do átrio esquerdo, mm	45,8 ± 8,5	48,1 ± 9,4	0,005
Número de comorbidades^†^	4 (3 - 5)	4 (2 - 5)	0,665
**Número de procedimentos cirúrgicos anteriores, n(%)** ^**‡**^			0,002
0	702 (67,5)	65 (55,6)	
1	288 (27,7)	36 (30,8)	
2	46 (4,4)	14 (12,0)	
3 ou mais	4 (0,3)	2 (1,7)	
**Características clínicas na admissão**			
**Perfil hemodinâmico, n(%)**			0,020
A	78 (7,5)	8 (6,8)	
B	797 (76,6)	77 (65,8)	
C	130 (12,5)	25 (21,4)	
L	35 (3,4)	7 (6,0)	
Pressão arterial sistólica, mmHg	123 ± 29	115± 27	0,004
Pressão arterial diastólica, mmHg	76 ± 17	71 ± 16	0,001
Baixa perfusão periférica, n(%)	127 (12,2)	20 (17,1)	0,175
Ortopneia, n(%)	757 (74,6)	78 (70,9)	0,471
Ascite, n(%)	242 (24,6)	39 (35,5)	0,019
B3, n(%)	103 (10,3)	13 (11,5)	0,806
Edema periférico, n(%)	779 (75,6)	99 (85,3)	0,025
**Resultados laboratoriais na admissão (níveis sanguíneos)**			
Creatinina, mg/dL	1,71 ± 1,51	1,93 ± 1,30	0,136
BUN, mg/dL	33,07 ± 22,8	46,0 ± 30,4	< 0,001
Sódio, mmol/L	136,6 ± 5,0	134,2 ± 6,9	< 0,001
Potássio, mmol/L	4,46 ± 0,77	4,66 ± 0,92	0,010
Hemoglobina, g/dL	12,6 ± 2,28	12,32 ± 2,4	0,207
**Questionário WHOQOL-BREF**			
Domínio da saúde física	50,4 ± 19,4	39,5 ±15,8	< 0,001
Domínio de relações sociais	64,2 ± 16,5	61,1 ± 17,3	0,059
Saúde ambiental	58,9 ± 12,6	54,5 ± 12,9	<0,001
Saúde psicológica	77,3 ± 17,9	68,8 ± 18,0	<0,001

Variáveis categóricas são apresentadas como n (%) e variáveis contínuas como média ± desvio padrão ou mediana (percentil 25 - percentil 75). IC: insuficiência cardíaca; FEVE: fração de ejeção do ventrículo esquerdo; WHOQOL-BREF: Versão resumida do questionário de qualidade de vida da Organização Mundial da Saúde; BUN: Nitrogênio ureico no sangue. † As comorbidades incluíram hipertensão, diabetes, dislipidemia, acidente vascular cerebral, doença carotídea, doença arterial coronariana, doença vascular periférica, infarto agudo do miocárdio, fibrilação atrial, doença de Chagas, doença cardíaca reumática, doença valvar, doença pulmonar obstrutiva crônica, apneia do sono, doença hepática, insuficiência renal crônica, anemia, hipertireoidismo, hipotireoidismo, depressão, câncer de órgão sólido e câncer hematológico. ‡ Procedimentos cirúrgicos anteriores incluíram angioplastia coronária, cirurgia de revascularização do miocárdio, marcapasso ou desfibrilador cardíaco implantável, terapia de ressincronização cardíaca e prótese valvar.

**Tabela 2:** – Comparação das variáveis utilizadas nos escores ADHERE, GWTG e ML-HF

VARIÁVEL	ADHERE	GWTG	ML-HF
**Demográfico**			
Idade, anos	✓	✓	
Raça negra		✓	
**Características clínicas**			
BUN, mg/dL	✓	✓	✓
Creatinina, mg/dL	✓		✓
Diâmetro do átrio esquerdo			✓
DPOC		✓	
Doença Renal Crônica			✓
Etiologia valvar da IC			✓
Frequência cardíaca		✓	
Frequência respiratória		✓	
Hemoglobina, g/dL			✓
Hipotireoidismo			✓
Número de procedimentos cardíacos anteriores			✓
Pressão arterial sistólica, mmHg	✓	✓	✓
Pressão Arterial Diastólica, mmHg			✓
Perfil hemodinâmico B			✓
Perfil hemodinâmico C			✓
Perfil hemodinâmico L			✓
Má perfusão periférica			✓
Prótese valvar			✓
Sódio, mmol/L		✓	✓
**Qualidade de vida**			
Domínio da saúde física – WHOQOL-BREF			✓

BUN: Nitrogênio ureico sanguíneo; DPOC: doença pulmonar obstrutiva crônica; WHOQOL-BREF: Versão resumida do questionário de qualidade de vida da Organização Mundial da Saúde.

### Seleção de algoritmo

Dentre os algoritmos de ML avaliados, o modelo Random Forest apresentou o melhor desempenho na predição de mortalidade hospitalar em comparação com Gradient Boosting Machines e Extreme Gradient Boosting ( Tabela 3 ). Dessa forma, o algoritmo Random Forest foi escolhido para construir o modelo final.

**Tabela 3:** – Desempenho de discriminação no conjunto de treinamento de acordo com o algoritmo de Aprendizado de Máquina

Algoritmo de Aprendizado de Máquina	AUC (Média ± DP) N = 810
Random Forest	0,772 ± 0,07
Gradient Boosting Machines	0,703 ± 0,08
Extreme Gradient Boosting	0,724 ± 0,09

DP: desvio padrão; AUC: área sob a curva de operação de recepção.

### Validação interna

**Figura 3:**
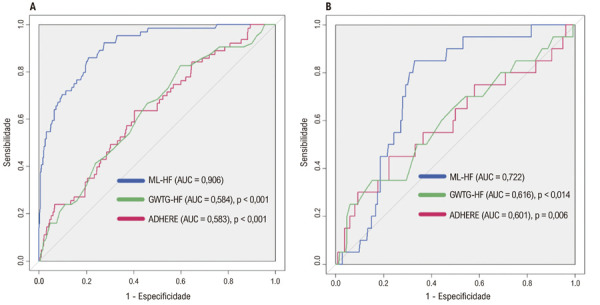
– Comparação das curvas ROC (Receiver Operating Characteristic) entre os escores ML-HF, GWTG-HF e ADHERE nos conjuntos de treinamento (A) e teste (B).

No conjunto de treinamento (n=621), o escore ML-HF demonstrou precisão de boa a excelente para prever mortalidade hospitalar, com uma AUC de 0,906, superando os escores ADHERE e GWTG-HF. [ Figura 3A ] [ Figura Central ].

### Validação externa

No conjunto de teste (n=536), o escore ML-HF manteve uma discriminação aceitável, com uma AUC de 0,722. Embora inferior ao conjunto de treinamento, seu desempenho permaneceu superior aos escores ADHERE e GWTG. [ Figura 3B , Figura Central]. Resultados semelhantes foram observados ao utilizar apenas a coorte 2 completa como conjunto de teste (n=280), conforme mostrado na Figura S2 Suplementar.

### Calibração do modelo

**Figura 4:**
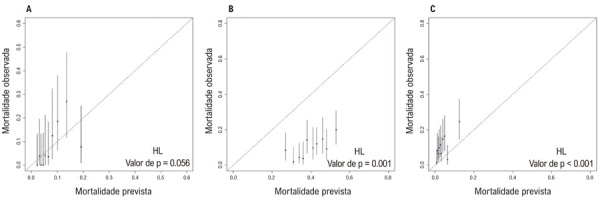
– Comparação da mortalidade hospitalar observada e prevista para avaliar a calibração do modelo dos escores ML-HF (A), GWTG-HF (B) e ADHERE (C) no conjunto de teste. HL: Teste de Hosmer-Lemeshow. Um valor de p menor que 0,05 indica um ajuste inadequado dos dados.

A Figura 4 mostra a mortalidade hospitalar observada versus prevista para cada escore no conjunto de teste. Embora o escore ML-HF tenha apresentado boa calibração do modelo, a mortalidade prevista divergiu significativamente da mortalidade observada nos outros dois escores convencionais. Especificamente, o escore ADHERE subestimou o risco, enquanto o escore GTWG o superestimou.

### Importância da variável no escore ML-HF

**Figura 5:**
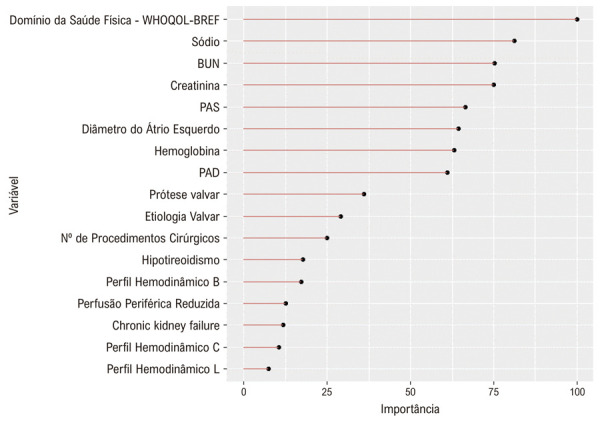
– Importância das variáveis incluídas no escore ML-HF. WHOQOL-BREF: Versão resumida do questionário de qualidade de vida da Organização Mundial da Saúde; PAS: pressão arterial sistólica; PAD: pressão arterial diastólica; BUN: Nitrogênio ureico no sangue.

A Figura 5 mostra a importância relativa das variáveis do escore ML-HF em ordem decrescente. A variável com maior importância foi o domínio Saúde Física do WHOQOL, seguido pelos níveis sanguíneos de sódio, ureia e creatinina, conforme mostrado na Figura Central. A pressão arterial na admissão também estava entre as dez variáveis mais importantes, com a pressão arterial sistólica e a pressão arterial diastólica ocupando a quinta e a oitava posições, respectivamente. Notavelmente, a FEVE não teve importância suficiente para predizer a mortalidade hospitalar no método de Boruta. O único parâmetro ecocardiográfico no modelo foi o tamanho do átrio esquerdo, ocupando o sexto lugar em ordem de importância.

## Discussão

Este estudo apresenta três principais achados. Desenvolvemos um novo escore baseado em ML – o escore ML-HF – para prever a mortalidade hospitalar em pacientes com ICA em uma PBMR, que foi validado externamente com acurácia aceitável e boa calibração. O escore ML-HF superou os escores convencionais ADHERE e GWTG-HF na mesma população.^2 - 5^ O estudo revelou que as informações coletadas a partir de um questionário de qualidade de vida emergiram como o preditor mais importante de mortalidade. No geral, este estudo fornece uma nova ferramenta para prever o risco em pacientes com IC aguda e destaca o potencial do ML para aprimorar os modelos de estratificação de risco nessa população.

Até onde sabemos, este é o primeiro estudo a incorporar uma medida de qualidade de vida pessoal em um modelo baseado em ML para prever mortalidade na IC. Surpreendentemente, o domínio saúde física do questionário WHOQOL-BREF emergiu como o preditor mais importante de mortalidade, superando biomarcadores séricos e parâmetros ecocardiográficos. Esse achado novo e inesperado provavelmente só foi possível graças ao uso de técnicas de ML, pois elas permitem a análise de variáveis de maneira não linear e predefinida. Este é um exemplo de como o ML pode analisar padrões complexos em dados para encontrar preditores contraintuitivos da perspectiva humana. Este resultado ressalta a importância de incorporar os resultados relatados pelo paciente (RRP) e considerar a autopercepção do paciente sobre sua saúde. Em termos mais simples, destaca a importância de realmente ouvir o paciente.

Estudos anteriores demonstraram o valor prognóstico do RRP em pacientes com IC crônica. No registro CHAMP-HF ( *Change the Management of Patients With Heart Failure* ), uma melhora de 5 pontos no Questionário de Cardiomiopatia de Kansas City foi associada a uma redução de 41% no risco de morte por todas as causas.^19^ Notavelmente, o RRP provou ser um melhor preditor de eventos futuros em comparação com a classe funcional da NYHA, ressaltando a importância de avaliar a perspectiva do paciente sobre seu estado de saúde, além da avaliação clínica.^19 , 20^ De fato, as diretrizes atuais de IC recomendam a avaliação padronizada do RRP como uma recomendação de classe 2a.^10^ Nosso estudo contribui para a literatura ao demonstrar o papel prognóstico independente da medição do RRP na admissão de pacientes com IC descompensada.

É possível destacar o fato de que novas variáveis, além do domínio de saúde física do WHOQOL-Brief, foram relevantes para a predição de óbito hospitalar em nosso estudo, fatores que não são avaliados em outros escores, como hemoglobina, diâmetro do átrio esquerdo, presença de prótese valvar, número de procedimentos cirúrgicos e presença de hipotireoidismo. A relevância da presença de prótese valvar pode ser devida à alta prevalência de febre reumática em nossa população. Isso, mais uma vez, destaca a importância do desenvolvimento de escores personalizados para PBMR.^21^

Modelos de ML foram testados anteriormente para prever a mortalidade hospitalar de pacientes com IC aguda em outros países. O modelo MARKER-HF ( *Machine learning Assessment of RisK and EaRly mortality in Heart Failure* ) demonstrou excelente poder discriminatório, com uma AUC de 0,88. No entanto, esse modelo foi desenvolvido usando pacientes com IC crônica e aguda, com durações de acompanhamento variadas.^9^ Segar et al. utilizaram dados do registro GWTG nos Estados Unidos para construir um modelo baseado em ML para prever a mortalidade hospitalar, que foi validado na coorte ARIC ( *Atherosclerosis Risk in Community* ).^5^ Assim como em nossa investigação, eles observaram que o modelo baseado em ML superou o escore GWTG-HF, apresentando um índice C de aproximadamente 0,80. Eles puderam incorporar biomarcadores séricos, como níveis sanguíneos de troponina e peptídeos natriuréticos, e determinantes sociais de saúde em nível contextual, o que ajuda a reduzir o viés do algoritmo e melhorar a equidade entre populações carentes.^4 , 5^

Diferenças nas populações de pacientes, no manejo clínico e na disponibilidade de biomarcadores, como peptídeos natriuréticos, podem explicar, em parte, a maior precisão do modelo de Segar et al.^5^ em comparação ao nosso; no entanto, nosso modelo pode ser particularmente valioso em cenários com acesso limitado a esses biomarcadores, especialmente em PBMR. Em nosso estudo, determinantes sociais individuais foram utilizados, mas não selecionados a partir do algoritmo baseado em ML. Por outro lado, demonstramos pela primeira vez que uma medida de qualidade de vida ajuda a melhorar a previsão de morte hospitalar de pacientes com IC em um modelo baseado em ML.

Nosso estudo tem pontos fortes que devem ser destacados. Primeiramente, analisamos os determinantes sociais da saúde individualmente, embora não tenham sido selecionados como variáveis importantes. Esse achado está em linha com a literatura atual, que afirma que os determinantes sociais podem ser relevantes apenas em subgrupos mais homogêneos, o que pode não ser o caso da população brasileira.^4^ Em segundo lugar, também realizamos validação externa usando uma coorte diferente daquela usada para o treinamento do modelo, o que deve ser incentivado em estudos que utilizam ML.^7^ Em terceiro lugar, nossa amostra contém pacientes de todas as macrorregiões do país, abrangendo uma ampla gama de características demográficas e status sociais. Essa diversidade aumenta a generalização e a validação de nossos achados em diferentes populações. Em quarto lugar, nossos dados foram coletados sistematicamente como parte de um estudo de coorte prospectivo, em vez de depender de RME. que são mais suscetíveis ao viés de classificação incorreta.^22 - 26^

Este estudo também apresenta limitações que devem ser consideradas. Dados ausentes levaram a uma redução no tamanho da amostra inicial, diminuindo-a para aproximadamente 25% do tamanho original, pois removemos pacientes com dados ausentes em qualquer uma das variáveis mais significativas. Incluímos apenas casos completos, assumindo que os dados estavam completamente ausentes aleatoriamente. Optamos por não imputar múltiplas variáveis para evitar a introdução de viés potencial de estimativas baseadas em modelos em um ambiente preditivo. Além disso, os peptídeos natriuréticos não foram utilizados para construir o modelo devido à falta de disponibilidade na maioria dos centros, por se tratar de um banco de dados em que a grande maioria dos locais é financiada publicamente. Reconhecemos que a ausência desses marcadores pode prejudicar a análise, visto que atualmente são testes essenciais para a confirmação do diagnóstico de IC. Em países com maior disponibilidade, acreditamos que essa variável pode ser ainda mais relevante e aumentar a precisão do algoritmo. Por outro lado, isso poderia melhorar a aplicabilidade do escore ML em outros PBMR, onde o acesso ao BNP também pode ser limitado.

Apesar dos resultados promissores, a implementação de escores de risco baseados em ML na prática clínica continua desafiadora. Ao contrário dos escores de risco tradicionais, que podem usar tabelas ou calculadoras simples, os modelos de ML exigem infraestrutura digital para entrada de dados, processamento e integração de resultados em fluxos de trabalho clínicos. Em nosso estudo, o desenvolvimento e a validação do modelo foram realizados em um computador centralizado contendo dados de todos os centros participantes, e a análise foi computacionalmente intensiva. Para uso no mundo real, a implantação exigiria sistemas compatíveis e entrada de dados padronizada. Estratégias como o desenvolvimento de aplicativos móveis offline ou a integração do modelo em prontuários eletrônicos de saúde de baixa complexidade poderiam ser exploradas para facilitar a implementação em ambientes com recursos limitados.

Embora o modelo atual possa ser aplicado sem retreinamento, os modelos baseados em ML podem ser atualizados ou retreinados periodicamente para melhorar o desempenho e se adaptar à evolução das populações clínicas. Isso levanta importantes considerações éticas e regulatórias, incluindo privacidade de dados, consentimento informado para uso secundário de dados de pacientes e transparência em relação aos procedimentos de atualização do modelo. A implementação eficaz dependerá não apenas de uma infraestrutura técnica adequada para atender às demandas computacionais, mas também de fortes estruturas de supervisão e governança para garantir o uso seguro, equitativo e responsável.

## Conclusão

Nós desenvolvemos e validamos um modelo baseado em aprendizado de máquina - o escore ML-HF - para prever o risco de morte hospitalar em pacientes com IC aguda. Essa ferramenta preditiva apresentou poder discriminativo superior aos escores tradicionais ADHERE e GWTG.

## *Material suplementar

Para informação adicional, por favor, clique aqui. https://abccardiol.org/supplementary-material/2025/12211/2025-0136_supplementary_material.pdf
