# Supplementary material for: Um Novo Escore de Risco Baseado em Aprendizado de Máquina (Machine Learning) em Pacientes com Insuficiência Cardíaca Aguda: O Escore ML-HF
Source: Arq Bras Cardiol. 2025 Dec 17;122(11):e20250136. [Article in Portuguese] doi: 10.36660/abc.20250136 (PMC12978276; doi:10.36660/abc.20250136)
Supplement: Supplementary file 1 [file 0066-782x-abc-122-11-e20250136-suppl01.pdf]

1. SUPPLEMENTARY MATERIAL

Table S1– Missing data rate at all variables

Variable	Nº of missing	Percentage (%)
BNP (pg/mL)	2917	95,86
E/e' ratio	2903	95,40
NTProBNP (pg/mL)	2629	86,40
Left atrium volume (mL)	2376	78,08
Diastolic dysfunction	2342	76,96
Pulmonary artery systolic pressure (mmHg)	2248	73,87
Fasting blood glucose (mg/dl)	2074	68,16
Troponin above superior value of normality	1710	56,19
QRS duration (ms)	1532	50,35
Self-Perception Domain WHOOQOL-Bref	971	31,91
Physical Health Domain WHOOQOL-Bref	969	31,84
Psychological health Domain WHOOQOL-Bref	969	31,84
Social Relationships Domain WHOOQOL-Bref	969	31,84
Environment Domain WHOOQOL-Bref	969	31,84
NYHA functional class	888	29,18
Body mass index (Kg/m^2)	793	26,06
Hemodynamic profile	776	25,50
Decreased appetite/ early satiety	603	19,82
Visual analog scale	580	19,06
Sinusal Rhythm	571	18,76
Left atrium diameter (mm)	537	17,65
Pathological jugular ingurgitation	463	15,22
Hepatomegaly	415	13,64
Volume overload/ weight gain	394	12,95
Ascites	389	12,78
Palpitation	348	11,44

Hisses	347	11,40
B3	345	11,34
Dizziness/ syncope	344	11,30
Low peripheral perfusion/circulatory shock	336	11,04
ICD/ Sustained ventricular arrhythmia shock	331	10,88
Nocturnal paroxysmal dyspnea	248	8,15
Dyspnea when climbing stairs	232	7,62
Chest pain	232	7,62
Orthopnea	199	6,54
Pulmonary rales	190	6,24
Dyspnea when walking on plane ground	188	6,18
Serum Potassium (mmol/l)	183	6,01
Serum Sodium (mmol/L)	170	5,59
Systolic blood pressure (mmHg)	167	5,49
Diastolic blood pressure (mmHg)	165	5,42
Serum Urea (mg/dL)	164	5,39
Cardiac rate (bpm)	157	5,16
Left Ventricular Ejection Fraction (%)	145	4,77
Hemoglobin (g/dL)	142	4,67
Carotid disease	141	4,63
Sleep apnea	139	4,57
Peripheral edema	137	4,50
Depression	135	4,44
Dyspnea at rest	134	4,40
Hematological cancer	121	3,98
Peripheral vascular disease	117	3,84
Solid organ cancer	117	3,84
Hyperthyroidism	110	3,61
Other risk factors and comorbidities	110	3,61

Hypotireodism	109	3,58
Chagas disease	108	3,55
Serum creatinine (mg/dL)	108	3,55
Cardiac rheumatic disease	107	3,52
Coronary artery disease	97	3,19
Liver disease	97	3,19
COPD	93	3,06
Dyslipidemia	92	3,02
Valve disease	87	2,86
Stroke	84	2,76
Anemia	83	2,73
Dialitic CKD	78	2,56
Acute myocardial infarction	77	2,53
Atrial flutter/Atrial fibrilation	73	2,40
Chronic kidney disease (Cr> 2.0)	71	2,33
Diabetes mellitus	48	1,58
Arterial hypertension	30	0,99
Previous Heart Failure	28	0,92
No symptoms	23	0,76
Pleural effusion	9	0,30
Pulmonary edema	9	0,30
Cardiothoracic index > 0.5	9	0,30
Redistribution of flow for upper lobes	9	0,30
Family income	6	0,20
Age	5	0,16
Cardiac Resynchronization Therapy	4	0,13
Coronary angioplasty	3	0,10
Level of education	2	0,07
Smoking status	2	0,07

Alcohol Consumption History	2	0,07
Use of illicit drugs	2	0,07
Regular physical exercise	2	0,07
Myocardial revascularization surgery	1	0,03
ICD	1	0,03
Sex	0	0,00
Race	0	0,00
Patient has any risk factor or other comorbidities beyond the motive for hospitalization?	0	0,00
Have a history of previous cardiological procedures?	0	0,00
Definitive Pacemaker	0	0,00
Valve prosthesis	0	0,00
Cardiac transplantation	0	0,00
Cardiac insufficiency	0	0,00
Unknown etiology	0	0,00
Arrhythmia	0	0,00
Respiratory complication	0	0,00
Renal decompensation	0	0,00
Non-adhesion to drug therapy	0	0,00
Infection	0	0,00
Ischemia	0	0,00
Unattended hypertension	0	0,00
Others etiologies	0	0,00
Nº of comorbidities	0	0,00
Nº of previous cardiac procedures	0	0,00
Nº of symptoms at admission	0	0,00
Nº of clinical signs at admission	0	0,00

*In red, the excluded variables with more than 40% missing data

BNP – B-type natriuretic peptide; NTProBNP – N-terminal B-type natriuretic peptide; WHOQOL-BREF – World Health Organization quality of life brief version; NYHA – New York Heart Association; ICD – Implantable cardioverter-defibrillator

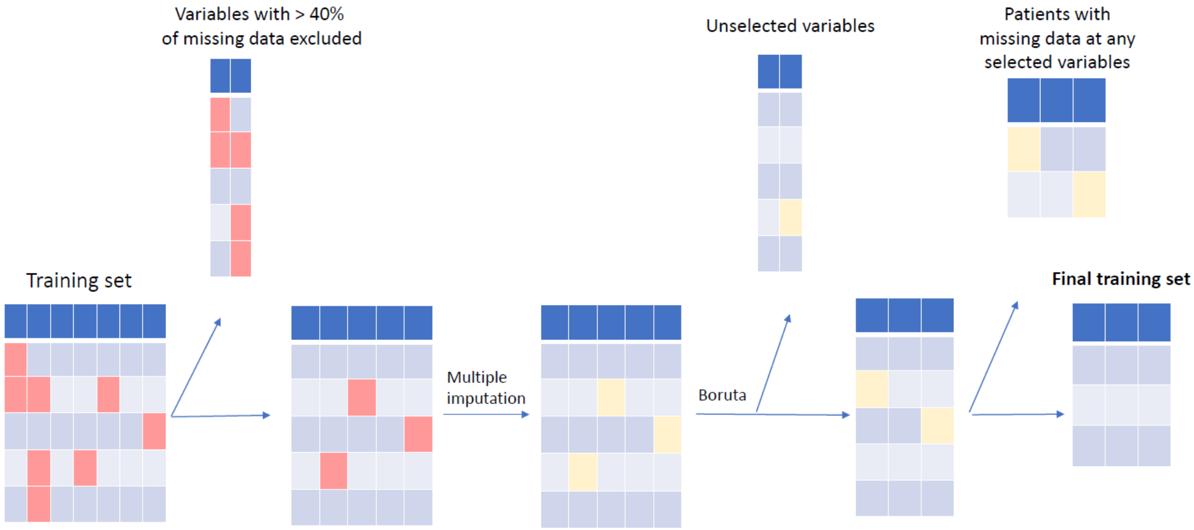

Figure S1 – Data treatment process

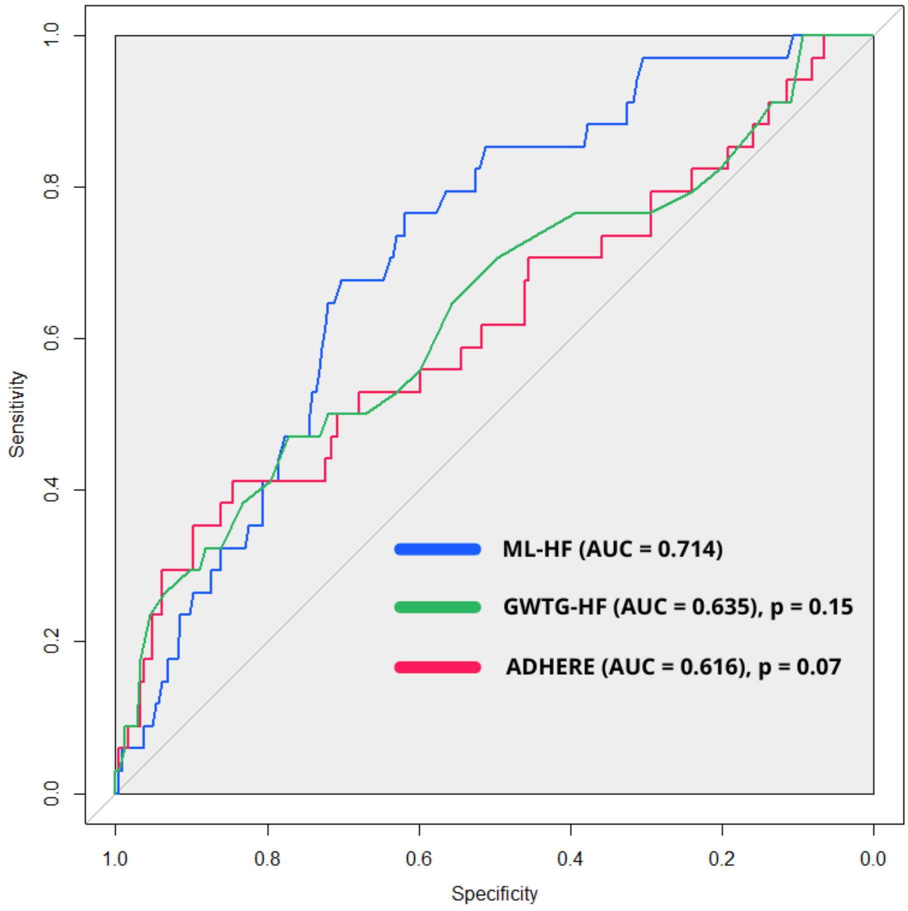

Figure S2 – Comparison of the receiver operating characteristic (ROC) curves among the three models in the full Cohort 2 only as the test set
