# Supplementary material for: Um Novo Escore de Risco Baseado em Aprendizado de Máquina (Machine Learning) em Pacientes com Insuficiência Cardíaca Aguda: O Escore ML-HF
Source: Arq Bras Cardiol. 2025 Dec 17;122(11):e20250136. [Article in Portuguese] doi: 10.36660/abc.20250136 (PMC12978276; doi:10.36660/abc.20250136)
Supplement: Translation

# Translation: A New Risk Score Based on Machine Learning in Patients with Acute Heart Failure: The ML-HF score

**DOI:** 10.36660/abc.20250136i

**Authors:** Matheus Bissa Duarte Ferreira, Jorge Tadashi Daikubara, Gustavo S. Pereira da Cunha, Rafael Moretti, Jessica Tamires Reichert, Lucas Müller Prado, Raphael Henrique Déa Cirino, Sidney C Smith, Fábio Papa Taniguchi, Andrei C. Sposito, Odilson M. Silvestre, Wilson Nadruz, Miguel Morita Fernandes-Silva

**Affiliations:** 1 Brazil Universidade Federal do Paraná, Curitiba, PR – Brazil; 2 USA University of North Carolina At Chapel Hill, Chapel Hill, North Carolina – USA; 3 Brazil Hospital do Coração (HCor), São Paulo, SP – Brazil; 4 Brazil Universidade Estadual de Campinas, Campinas, SP – Brazil; 5 Brazil Universidade Federal do Acre, Rio Branco, AC – Brazil

**Keywords:** Heart Failure, Machine Learning, Death

## Abstract

**Background:**

Conventional prognostic assessment scores often fall short in performance to predict mortality in patients with acute heart failure (AHF).

**Objective:**

To develop and validate a machine learning-based prognostic score to predict in-hospital death in patients with AHF and compare its performance with the main traditional scores.

**Methods:**

Patients admitted for AHF in Brazilian hospitals from the “Best Practices in Cardiology Program” from 2016 - 2022 were included. Clinical data, laboratory results, and the World Health Organization Quality of Life (WHOQOL-Bref) at hospital admission were collected. The outcome was in-hospital death. The model was trained using 70% of admissions (training set) and validated with the remaining 30% (test set). The ML-HF score area under the ROC curve (AUC) was compared with the AUC of the traditional scores Acute Decompensated Heart Failure National Registry (ADHERE) and Get With the Guidelines–Heart Failure (GWTG-HF). The level of significance was p<0.05.

**Results:**

One thousand and one hundred fifty-seven patients hospitalized for AHF were included. The five most important variables of the ML-HF score were: Physical Health Domain Quality (WHOQOL-BREF), serum sodium, serum urea, serum creatinine, and systolic blood pressure at hospital admission. In the test set, the ML-HF score showed an adequate model calibration (Hosmer-Lemeshow test p value=0.056) and discrimination (AUC=0.722 [CI95%, 0.661-0.783]), which was superior to GWTG-HF (AUC=0.616 [CI95%, 0.529-0.702; p=0.014]) and the ADHERE (AUC=0.601 [CI95%, 0.511-0.691; p=0.006]) scores.

**Conclusion:**

We developed and validated a score using machine learning to predict in-hospital death in patients with AHF, which outperformed the traditional scores.

## Introduction

Heart failure (HF) affects 25 million people worldwide and has high mortality and hospitalization rates. In Brazil, there are approximately 250,000 hospital admissions due to HF per year, resulting in a 10.9% in-hospital mortality rate.^1^

Risk classification tools help direct resources and intensive measures to higher-risk patients. However, estimating the prognosis of patients upon admission or during hospitalization has been challenging in the HF scenario. Previous studies have developed scores, such as the Acute Decompensated Heart Failure National Registry (ADHERE) and the Get With The Guidelines-Heart Failure (GWTG - HF) scores, which can be derived from a few laboratory and clinical variables, to predict in-hospital mortality in these patients.^2 , 3^Unfortunately, these scores have sub-optimal accuracy and have been underused in clinical practice.^4 , 5^

An alternative to these conventional approaches are the models based on Machine Learning (ML), which have been studied in the last years for the management of HF.^6 , 7^The ability of ML algorithms to analyze in a deeper, multi-dimensional, and non-linear way a variety of variables increases their potential to improve patient stratification models.^7^ML-based models have been built in patients with chronic and acute HF (AHF) using electronic medical records (EMR), registries, and clinical trials.^8^These models appear to have superior accuracy than the traditional ADHERE and GWTG-HF scores.^9^However, most of these studies have been developed and validated in high-income countries. Differences in patient population, social determinants, and healthcare delivery may prevent these models from being implemented in low- and middle-income countries (LMIC). In fact, it has already been shown that the ability of ML models to predict HF outcomes significantly changes across different ethnic and socioeconomic subgroups.^10^

Therefore, we developed and validated an algorithm based on ML to predict in-hospital deaths in patients with AHF using a large cohort study of public hospitals in Brazil. We further compared the final ML-based algorithm with the ADHERE and GWTG models.

## Methods

### Population

This is an analysis of the "Best Practice in Cardiology (BPC)" program, which was adapted from the "Get With The Guidelines" (GWTG) initiative to improve the quality of cardiovascular care and patients' outcomes in acute coronary syndrome, HF, and atrial fibrillation in Brazilian public hospitals. The study design, rationale, procedures, and main results have been previously published.^11^Briefly, tertiary public hospitals in all five Brazilian macro-regions have been selected since January 2016.^12^These hospitals were included in a quasi-experimental study which implemented an institutional-level intervention to improve adherence to guidelines and evaluated its impact on measures of institutional performance. Sociodemographic, clinical, laboratory, and echocardiographic data were collected during patient admission. Patients were also asked to complete the World Health Organization Quality Of Life (WHOQOL-Bref) questionnaire, which is a 26-item instrument consisting of four domains: physical health (7 items), psychological health (6 items), social relationships (3 items), and environmental health (8 items). The items of the WHOQOL-Bref consist of 5-point Likert scales that are combined to provide each health domain. The procedure to calculate the WHOLQOL-BREF was described in detail elsewhere.^13^As part of the BPC program, all participants were followed up with visits at discharge, one month, and six-month. This study was approved by the ethics committee of the Coordinating Center (number 48561715.5.1001.0060) and of each participating hospital. All participants gave written informed consent for participation in the study.

### Inclusion and exclusion criteria

For this analysis, we included patients aged 18 years or older hospitalized with a primary diagnosis of AHF (ICD-10 codes I50; I50.0; I50.1 or I50.9) between 14/03/2016 and 07/12/2022 in any participating hospital of the BPC program, who agreed to participate in the study and signed the informed consent form as illustrated in the Central Illustration . Participants were required to have been hospitalized for at least 24 hours to be eligible.

We excluded patients transferred to another hospital or with missing vital status at discharge. Patients who were on the waiting list for a heart transplant or using a ventricular assist device were also excluded. We also excluded patients with missing data for the selected variables of the model (see variable selection below).

To build and validate the model, we divided the dataset into 2 Cohorts: Cohort 1, patients admitted from 16/01/2016 to 15/10/2021, and Cohort 2, patients admitted from 16/10/2021 to 07/12/2022. Cohort 1 was used in the variable selection, model building, and validation processes. Cohort 2 was used only for external validation (see Statistical analysis for details).

### Outcome

The outcome was death from any cause during the index hospitalization.

#### Variable selection

We used all variables at admission in the predictive model, which included sociodemographic, clinical, laboratory, and echocardiographic data, along with the scores derived from the responses of the WHOQOL-Bref questionnaire. First, we excluded variables with more than 40% of data missing.^14^See supplemental table S1 for all variables and respective missing data rates. Then, we applied the ML method "Boruta" in the training set (see below) to automatically detect the most relevant variables that were selected for the training algorithm. The Boruta method generates a binomial probability distribution of the number of times a variable exceeds the importance of a created dummy variable, which has a random (non-predictive) value.^15^Variables were selected if they had a p-value < 0.05 from the upper tail of the probability distribution. During this selection process, we applied a K-Nearest Neighbor imputation method, selecting the K nearest (K = 5) neighbors with complete data based on a Gower distance and inputting the mean value of the complete variable to the neighbors with missing values.^16^

After selecting the most relevant variables, they were used to build the ML algorithm in the non-imputed database. Figure S1 illustrates the variable selection process ( Supplementary Figure S1 ). We conducted a Complete Case Analysis, including only patients with complete data for the selected variables. Thus, patients with missing data for any of the selected variables were excluded ( Figure 1 ).

**Figure 1:** – Flowchart of patients included in the analysis.

## Statistical analysis

### Summary Statistics

Continuous variables were assessed for normality through visual inspection of histograms, skewness, and kurtosis, and by applying the Kolmogorov-Smirnov test. Data are presented as mean ± standard deviation or median (25^th^percentile - 75^th^percentile), as appropriate. Between-group comparisons were performed using the unpaired Student's t-test for normally distributed variables and the Mann-Whitney U test for non-normally distributed variables. Categorical variables were expressed as counts and percentages and compared using Fisher's exact test.

### Model development and validation

Cohort 1 was randomly divided into training (70%) and test (30%) sets. This procedure was performed using a computer-generated random sampling without replacement, ensuring equal probability of selection of each patient. The training set was used for model training and internal validation. The test set combined 30% of Cohort 1 with the full Cohort 2 and was used for external validation. A sensitivity analysis was performed using only Cohort 2 as the test set. This process is illustrated in Figure 2 .

**Figure 2:** – Flowchart illustrating the internal and external validation of the ML-HF score model.

### Model selection

We employed a 5-fold cross-validation method to select the most suitable ML algorithm. This method consists of subsetting the training set into 5 random groups. Four of these subsets were used to train the model, and the remaining subset to test it. It is an iterative process that occurs 5 times, changing the test subset, as illustrated in Figure 2 . The algorithm with the best mean AUC was chosen as the final model.

### Model performance

To evaluate model discrimination, we constructed the receiver-operating characteristic (ROC) curves and compared the area under the ROC curve (AUC) using in-hospital death as the outcome. Model calibration was evaluated by plotting the predicted versus observed probability of in-hospital death across deciles and calculating the Hosmer-Lemeshow goodness-of-fit test. A p-value < 0.05 indicated inadequate fit of the data, while higher p-values indicated adequate calibration.

### Comparison with traditional scores

The ADHERE and GWTG-HF scores were calculated for each patient. Since the GWTG-HF score does not directly output the probabilities of death, we derived a logistic regression of its numeric outputs.^2 , 3^We compared the AUC-ROC between scores using DeLong's test in both training and test sets.^17^

We followed the Transparent Reporting of a Multivariable Prediction Model for Individual Prognosis or Diagnosis (TRIPOD) guideline for conducting our research and writing this article.^18^

All analyses and model training were conducted using R software. We considered statistically significant p-values < 0.05.

## Results

From January 2016 to December 2022, a total of 3,532 patients with AHF were included in the BPC program. After excluding those with VAD, on the transplant waiting list, or with missing vital status at discharge, 3,043 were considered eligible for the study (2,309 in Cohort 1 and 734 in Cohort 2, as seen in Figure 1 ).

Of 113 variables available at admission, nine were excluded due to more than 40% missing data (Supplemental Table S1). The Boruta algorithm was applied to 70% patients (n=1,616) randomly selected from Cohort 1, which yielded 17 variables that were selected to build the ML model.

Out of the 3,043 eligible patients, 1,157 patients were included for the final analysis due to complete data for all 17 variables. The training set consisted of 621 patients, which accounted for 70% of the 879 individuals in Cohort 1, while the test set consisted of 536 patients, comprising 258 remaining patients of Cohort 1 and an additional 278 patients from Cohort 2.

The in-hospital mortality rate was 10.1%, with a total of 117 deaths among the 1,157 patients examined. The mean age of these patients was 61 ± 15 years, LVEF was 42 ± 17 % and 58% were male ( Table 1 ). Table 2 displays these selected variables along with variables used in the ADHERE and GWTG scores for comparison.

**Table 1:** – Baseline characteristics of included patients

Parameters	Discharged alive (n=1040)	In-hospital death (n=117)	p-value
**Age, years**	61.3 ± 14.5	61.6 ± 14.4	0.829
**Female sex, n (%)**	424 (40.8)	53 (45.3)	0.398
**White race, n(%)**	674 (64.8)	78 (66.7)	0.766
**Education, n(%)**			
Illiterate	84 (8.1)	9 (7.7)	0.242
Incomplete elementary school	399 (38.4)	47 (40.2)	
Elementary school	148 (14.2)	26 (22.2)	
Incomplete high school	72 (6.9)	8 (6.8)	
High school	225 (21.6)	20 (17.1)	
Incomplete higher education	30 (2.9)	2 (1.7)	
Complete higher education	82 (7.9)	5 (4.3)	
**Average income, n(%)**			
≤ 1 minimum wage	721 (69.3)	97 (82.9)	0.009
**Smoker, n(%)**			
No	520 (50)	56 (47.9)	0.889
Current	438 (42.1)	52 (44.4)	
Past	82 (7.9)	9 (7.7)	
**Alcoholic, n(%)**			
No	654 (62.9)	80 (68.4)	0.200
Current	246 (23.7)	28 (23.9)	
Past	140 (13.5)	9 (7.7)	
**HF etiology, n(%)**			
Ischemic	200 (19.2)	22 (18.8)	< 0.001
Chagas disease	95 (9.1)	13 (11.1)	
Valvar disease	123 (11.8)	30 (25.6)	
Other	622 (59.8)	52 (44.4)	
Valvar prosthesis, n(%)	338 (32.5)	52 (44.4)	0.013
Chronic kidney failure, n(%)	152 (14.6)	27 (23.1)	0.024
Hypothyroidism, n(%)	77 (7.4)	19 (16.2)	0.002
LVEF, %	41.7 ± 17.1	41.90 ± 18.5	0.911
Left atrium diameter, mm	45.8 ± 8.5	48.1 ± 9.4	0.005
Number of comorbidities†	4 (3 - 5)	4 (2 - 5)	0.665
**Number of previous surgical procedures, n(%)** ^**‡**^			0.002
0	702 (67.5)	65 (55.6)	
1	288 (27.7)	36 (30.8)	
2	46 (4.4)	14 (12.0)	
3 or more	4 (0.3)	2 (1.7)	
**Clinical characteristics at admission**			
**Hemodynamic profile, n(%)**			0.020
A	78 (7.5)	8 (6.8)	
B	797 (76.6)	77 (65.8)	
C	130 (12.5)	25 (21.4)	
L	35 (3.4)	7 (6.0)	
Systolic blood pressure, mmHg	123 ± 29	115± 27	0.004
Diastolic blood pressure, mmHg	76 ± 17	71 ± 16	0.001
Low peripheral perfusion, n(%)	127 (12.2)	20 (17.1)	0.175
Orthopnea, n(%)	757 (74.6)	78 (70.9)	0.471
Ascites, n(%)	242 (24.6)	39 (35.5)	0.019
B3, n(%)	103 (10.3)	13 (11.5)	0.806
Peripheral edema, n(%)	779 (75.6)	99 (85.3)	0.025
**Laboratory results at admission (blood levels)**			
Creatinine, mg/dL	1.71 ± 1.51	1.93 ± 1.30	0.136
BUN, mg/dL	33.07 ± 22.8	46.0 ± 30.4	< 0.001
Sodium, mmol/L	136.6 ± 5.0	134.2 ± 6.9	< 0.001
Potassium, mmol/L	4.46 ± 0.77	4.66 ± 0.92	0.010
Hemoglobin, g/dL	12.6 ± 2.28	12.32 ± 2.4	0.207
**WHOQOL-BREF questionnaire**			
Physical health domain	50.4 ± 19.4	39.5 ±15.8	< 0.001
Social relationships domain	64.2 ± 16.5	61.1 ± 17.3	0.059
Environmental health	58.9 ± 12.6	54.5 ± 12.9	<0.001
Psychological health	77.3 ± 17.9	68.8 ± 18.0	<0.001

Categorical variables are shown as n (%) and continuous variables as mean ± standard deviation or median (25^th^percentile - 75^th^percentile). HF: heart failure; LVEF: left ventricular ejection fraction; WHOQOL-BREF: World Health Organization quality of life brief version; BUN: Blood Urea Nitrogen. † Comorbidities included hypertension, diabetes, dyslipidemia, cerebrovascular accident, carotid disease, coronary artery disease, peripheral vascular disease, acute myocardial infarction, atrial fibrillation, Chagas disease, rheumatic heart disease, valve disease, chronic obstructive pulmonary disease, sleep apnea, liver disease, chronic kidney failure, anemia, hyperthyroidism, hypothyroidism, depression, solid organ cancer, and hematologic cancer. ‡ Previous surgical procedures included coronary angioplasty, myocardial revascularization surgery, pacemaker or implantable cardiac defibrillator, cardiac resynchronization therapy, and valve prosthesis.

**Table 2:** – Comparison of variables used in the ADHERE, GWTG, and ML-HF scores

VARIABLE	ADHERE	GWTG	ML-HF
**Demographic**			
Age, years	✓	✓	
Black race		✓	
**Clinical characteristics**			
BUN, mg/dL	✓	✓	✓
Creatinine, mg/dL	✓		✓
Left atrium diameter			✓
COPD		✓	
Chronic Kidney Disease			✓
Valve etiology of HF			✓
Heart rate		✓	
Respiratory frequency		✓	
Hemoglobin, g/dL			✓
Hypothyroidism			✓
Number of previous cardiac procedures			✓
Systolic Blood Pressure, mmHg	✓	✓	✓
Diastolic Blood Pressure, mmHg			✓
Hemodynamic profile B			✓
Hemodynamic profile C			✓
Hemodynamic profile L			✓
Bad peripheral perfusion			✓
Valve prosthesis			✓
Sodium, mmol/L		✓	✓
**Quality of life**			
Physical health domain – WHOQOL-BREF			✓

BUN: Blood urea nitrogen; COPD: chronic obstructive pulmonary disease; WHOQOL-BREF: World Health Organization quality of life brief version.

### Algorithm selection

Among the ML algorithms evaluated, the Random Forest model showed the highest performance in predicting in-hospital mortality compared to Gradient Boosting Machines and Extreme Gradient Boosting ( Table 3 ). Accordingly, the Random Forest algorithm was chosen to build the final model.

**Table 3:** – Discrimination performance in the training set according to the Machine Learning algorithm

Machine Learning Algorithm	AUC (Mean ± SD) N = 810
Random Forest	0.772 ± 0.07
Gradient Boosting Machines	0.703 ± 0.08
Extreme Gradient Boosting	0.724 ± 0.09

SD: standard deviation; AUC: area under the receiving operating curve.

### Internal validation

In the training set (n=621), the ML-HF score demonstrated good to excellent accuracy for predicting in-hospital mortality, with an AUC of 0.906, outperforming both the ADHERE and GWTG-HF scores. [ Figure 3A ] [ Central Illustration ].

**Figure 3:** – Comparison of the receiver operating characteristic (ROC) curves among the ML-HF, GWTG-HF, and ADHERE scores in the training (A) and testing (B) sets.

### External validation

In the test set (n=536), the ML-HF score maintained acceptable discrimination, with an AUC of 0.722. Although lower than in the training set, its performance remained superior to the ADHERE and GWTG scores ( Figure 3B , Central Illustration).. Similar results were observed when using the full Cohort 2 alone as the test set (n=280), as shown in Supplemental Figure S2.

### Model calibration

Figure 4 displays the observed vs predicted in-hospital mortality of each score in the test set. While the ML-HF score showed good model calibration, predicted mortality significantly diverged from the observed mortality in the other two conventional scores. Specifically, the ADHERE score underestimated the risk, while the GTWG score overestimated it.

**Figure 4:** – Comparison of observed and predicted in-hospital mortality to assess model calibration of the ML-HF (A), GWTG-HF (B), and ADHERE (C) scores in the testing set. HL: Hosmer-Lemeshow test. A p-value less than 0.05 indicates an inadequate fit of the data.

### Variable importance in ML-HF score

Figure 5 shows the relative importance of the ML-HF score variables in decreasing order. The variable with the highest importance was the Physical Health domain of the WHOQOL, followed by the sodium, BUN, and creatinine blood levels, as showed in the Central Illustration. Blood pressure at admission was also among the ten most important variables, with SBP and DBP ranking in the fifth and eighth positions, respectively. Notably, LVEF did not have sufficient importance to predict in-hospital mortality in the Boruta method. The only echocardiographic parameter in the model was left atrium size, ranking sixth in order of importance.

**Figure 5:** – Importance of the variables included in the ML-HF score. WHOQOL-BREF: World Health Organization quality of life brief version; SBP: systolic blood pressure; DBP: diastolic blood pressure; BUN: Blood Urea Nitrogen..

## Discussion

This study has three main findings. We developed a new ML-based score – the ML-HF score - to predict in-hospital mortality in patients with AHF in a LMIC, which was externally validated with acceptable accuracy and good calibration. The ML-HF score outperformed the conventional ADHERE and GWTG-HF scores in the same population.^2 - 5^The study revealed that information gathered from a quality-of-life questionnaire emerged as the most crucial predictor of mortality. Overall, this study provides a new tool to predict risk in AHF patients and highlights the potential of ML to improve risk stratification models within this population.

To our knowledge, this is the first study to incorporate a personal quality-of-life measure in an ML-based model to predict mortality in HF. Surprisingly, the physical health domain of the WHOQOL-BREF questionnaire emerged as the most important predictor of mortality, surpassing serum biomarkers and echocardiographic parameters. This new and unexpected finding was likely made possible only through the use of ML techniques, as they allow for the analysis of variables in a non-linear and predefined manner. This is an example of how ML can analyze intricate patterns in data to find counterintuitive predictors from the human perspective. This result underscores the importance of incorporating patient-reported outcomes (PRO) and considering the patient's self-perception about their health. In simpler terms, it highlights the value of truly listening to the patient.

Previous studies have shown the prognosis value of PRO in patients with chronic HF. In the CHAMP-HF (Change the Management of Patients With Heart Failure) registry, an improvement of 5 points in the Kansas City Cardiomyopathy Questionnaire was associated with a 41% lower risk of all-cause death.^19^Notably, PRO has proven to be a better predictor of future events compared to the NYHA functional class, underscoring the importance of assessing the patient's perspective on their health status in addition to the clinician assessment.^19 , 20^In fact, current HF guidelines recommend standardized assessment of PRO as a class 2a recommendation.^10^Our study adds to the literature by demonstrating the independent prognostic role of measuring PRO at the admission of patients experiencing decompensated HF.

It is possible to highlight the fact that new variables, in addition to the WHOQOL-Brief physical health domain, were relevant for predicting in-hospital death in our study, factors that are not assessed in other scores, such as hemoglobin, left atrium diameter, presence of prosthetic valve, number of surgical procedures, and presence of hypothyroidism. The relevance of the presence of a prosthetic valve may be due to the high prevalence of rheumatic fever in our population. This once again highlights the importance of developing personalized scores for LMIC countries.^21^

Machine-learning models have been previously tested to predict in-hospital mortality of patients with AHF in other countries. The MARKER-HF (Machine learning Assessment of RisK and EaRly mortality in Heart Failure) model demonstrated excellent discriminatory power, with an AUC of 0.88. However, this model was developed using both chronic and AHF patients, with varying follow-up durations.^9^Segar et al. utilized data from the GWTG registry in the United States to construct an ML-based model to predict in-hospital mortality, which was validated in the Atherosclerosis Risk in Community (ARIC) cohort.^5^Much like our investigation, they observed that the ML-based model outperformed the GWTG-HF score, boasting a C-index of approximately 0.80. They were able to incorporate serum biomarkers, such as troponin and natriuretic peptides blood levels, and contextual-level social determinants of health, which helps reduce algorithm bias and improve fairness among underserved populations.^4 , 5^

Differences in patient populations, clinical management, and availability of biomarkers such as natriuretic peptides may partly explain the higher accuracy of the Segar et al.^5^model compared to ours; however, our model may be particularly valuable in settings with limited access to these biomarkers, especially in LMIC. In our study, individual-level social determinants were used but not selected from the ML-based algorithm. On the other hand, we have shown for the first time that a quality-of-life measure helps improve the prediction of in-hospital death of HF patients in an ML-based model.

Our study has strengths that should be highlighted. Firstly, we analyzed social determinants of health individually, even though they were not selected as important variables. This finding is in line with current literature, which states that social determinants may be relevant only in more homogeneous subgroups, which may not be the case for the Brazilian population.^4^Secondly, we also performed external validation using a different cohort from that used for model training, which should be encouraged in studies using ML.^7^Thirdly, our sample contains patients from all macro regions of the country, encompassing a wide range of demographic characteristics and social statuses. This diversity enhances the generalizability and validation of our findings across different populations. Fourthly, our data was systematically collected as part of a prospective cohort study, rather than relying on EMR, which are more susceptible to misclassification bias.^22 - 26^

This study also has limitations that should be considered. Missing data led to a reduction in the initial sample size, decreasing it to approximately 25% of the original size as we removed patients with missing data in any of the most significant variables. We included only complete cases, assuming data was missing completely at random (MCAR). We opted against multiple imputations to avoid introducing potential bias from model-driven estimates in a predictive setting. In addition, natriuretic peptides were not used to build the model due to a lack of availability in most centers, because it is a database in which the vast majority of sites are publicly funded. We recognize that the absence of these markers may impair the analysis, as these are currently essential tests for confirming the diagnosis of HF. In countries with greater availability, we believe that this variable may be even more relevant and increase the accuracy of the algorithm. On the other hand, this could improve the applicability of the ML score in other LMIC, where access to BNP may also be limited.

Despite promising results, implementing ML-based risk scores in clinical practice remains challenging. Unlike traditional risk scores, which can use simple tables or calculators, ML models require digital infrastructure for data input, processing, and result integration into clinical workflows. In our study, model development and validation were performed on a centralized computer containing data from all participating centers, and analysis was computationally intensive. For real-world use, deployment would require compatible systems and standardized input data. Strategies such as developing offline mobile applications or integrating the model into low-complexity electronic health records could be explored to facilitate implementation in resource-limited settings.

While the current model can be applied without retraining, ML-based models may be periodically updated or retrained to improve performance and adapt to evolving clinical populations. This raises important ethical and regulatory considerations, including data privacy, informed consent for secondary use of patient data, and transparency around model updating procedures. Effective implementation will depend not only on adequate technical infrastructure to support computational demands but also on strong oversight and governance frameworks to ensure safe, equitable, and responsible use.

## Conclusion

We developed and validated a machine learning-based model - the ML-HF score - to predict in-hospital mortality among patients with AHF. This predictive tool presented a discriminative power superior to traditional ADHERE and GWTG scores.

## *Supplemental Materials

For additional information, please click here. https://abccardiol.org/supplementary-material/2025/12211/2025-0136_supplementary_material.pdf
